# Prevalence, quantitative load and genetic diversity of *Campylobacter* spp. in dairy cattle herds in Lithuania

**DOI:** 10.1186/1751-0147-55-87

**Published:** 2013-12-05

**Authors:** Sigita Ramonaitė, Anita Rokaitytė, Eglė Tamulevičienė, Alvydas Malakauskas, Thomas Alter, Mindaugas Malakauskas

**Affiliations:** 1 Department of Food Safety and Quality, Faculty of Veterinary Medicine, Veterinary Academy, Lithuanian University of Health Sciences, Tilzes 18, Kaunas LT-47181, Lithuania; 2 Clinic of Children Diseases, Medicine Academy, Lithuanian University of Health Sciences, A. Mickeviciaus 9, Kaunas LT-44307, Lithuania; 3 Department of Infectious Diseases, Faculty of Veterinary Medicine, Veterinary Academy, Lithuanian University of Health Sciences, Tilzes 18, Kaunas LT-47181, Lithuania; 4 Institute of Food Hygiene, Freie Universität Berlin, Königsweg 69, Berlin 14163, Germany

**Keywords:** Calves, Heifers, Cows, *Campylobacter* spp, Prevalence, Genetic diversity

## Abstract

**Background:**

Campylobacteriosis is a zoonotic disease, and animals such as poultry, pigs and cattle may act as reservoirs for *Campylobacter* spp. Cattle shed *Campylobacter* spp. into the environment and they can act as a reservoir for human infection directly via contact with cattle or their faeces or indirectly by consumption of contaminated food. The aim of this study was to determine the prevalence, the quantitative load and the genetic strain diversity of *Campylobacter* spp. in dairy cattle of different age groups.

**Results:**

Faecal samples of 200 dairy cattle from three farms in the central part of Lithuania were collected and examined for *Campylobacter*. Cattle herds of all three farms were *Campylobacter* spp. positive, with a prevalence ranging from 75% (farm I), 77.5% (farm II) to 83.3% (farm III). Overall, the highest prevalence was detected in calves (86.5%) and heifers (86.2%). In contrast, the lowest *Campylobacter* prevalence was detectable in dairy cows (60.6%). *C. jejuni*, *C. coli*, *C. lari* and *C. fetus* subsp. *fetus* were identified in faecal samples of dairy cattle. *C. upsaliensis* was not detectable in any sample. The high counts of *Campylobacter* spp. were observed in faecal material of dairy cattle (average 4.5 log_10_ cfu/g). The highest numbers of *Campylobacter* spp. were found in faecal samples from calves (average 5.3 log_10_ cfu/g), whereas, faecal samples from cows harboured the lowest number of *Campylobacter* spp. (average 3.7 log_10_ cfu/g). Genotyping by *fla*A PCR-RFLP analysis of selected *C. jejuni* isolates showed that some genotypes were present in all farms and all age groups. However, farm or age specific genotypes were also identified.

**Conclusions:**

Future studies are needed to investigate risk factors related to the degree of colonisation in cattle. Based on that, possible measures to reduce the colonisation and subsequent shedding of *Campylobacter* in cattle could be established. It is important to further investigate the epidemiology of *Campylobacter* in the cattle population in order to assess associated risks to public health.

## Background

*Campylobacters* are generally regarded as the most common bacterial cause of human gastroenteritis worldwide [1,2] and the species *C. jejuni* is responsible for 80% to 93.4% of the human campylobacteriosis cases depending on different geographic areas [3,4].

Several studies revealed that ruminants may play an important role in the epidemiology of this zoonosis [5,6]. Source attribution models attributed between 18%-38% of clinical strains or human cases to ruminant sources [7,8]. This is not surprising since up to 80% of cattle herds and 40–60% of the individual animals can shed *Campylobacter* spp. bacteria [9-11]. Despite the fact that consumption of contaminated poultry meat is assumed to be one of the most common cause of human campylobacteriosis [2], *C. jejuni* is frequently isolated from cattle of different ages as asymptomatic carriers of this pathogenic bacteria [9,12-14]. Proper application of biosecurity measures can lead to reduced colonization in poultry. However, biosecurity measures alone cannot to solve the problem. So far no intervention measure is available to effectively eradicate, prevent or reduce *Campylobacter* colonisation in primary animal production chain, including broiler production [15,16].

The humans could be infected with campylobacter from eating or drinking contaminated food, water, unpasteurized or raw milk or from close contact with infected animals. The consumption of unpasteurized milk has been the most important source of campylobacteriosis outbreaks [17]. Longer life span of dairy cattle than beef cattle can lead to permanent or long-term shedding of campylobacters by dairy cattle and these cattle serve as a long-term reservoir [18]. In addition, indirect exposure to cattle faeces through environmental contamination is considered a high risk to humans [19-21]. Up to now, there is limited and controversial information on the influence of the age of cattle on the *Campylobacter* prevalence [6,12,14,22,23].

Consequently, the role of different age groups of cattle from dairy farms as reservoir of *Campylobacter* spp. might be important for understanding the epidemiology of these pathogens.

The aim of this study was to evaluate the prevalence, the quantitative load and the genetic diversity of *Campylobacter* spp. in different age groups of cattle from dairy farms in the central part of Lithuania.

## Materials and methods

The research program for this study was approved by the Committee of the Veterinary Medicine and Zootechnics Sciences Areas (Protocol No.04/2010).

### Study design

Three dairy cattle farms (I, II, and III) with animal number on farms varying from 820 up to 1500 were included in the study. Rectal content grab samples were collected from May to August in 2012. All animals included in the study were clinically healthy. For each farm, animals were divided into three groups, depending on the age: calves (1–3 month of age), heifers (4–12 month of age) and cows (13–84 month of age). Altogether, 59 calves (farm I – 19, farm II - 20, farm II - 20), 80 heifers (farm I – 20, farm II - 40, farm II - 20) and 61 cow (farm I – 21, farm II - 20, farm II - 20) faecal samples were collected and tested for *Campylobacter* spp. For faecal sampling all farms were visited twice. On all farms, milking cows were housed inside throughout the year without access to pastures. Heifers were kept in groups of 10–20 animals per group and had access to outside areas in all farms. Calves ware kept in individual pens until the age of 5–15 days. After that, they were regrouped into groups of 10–15 animals until the age of 3 months. In contrast, calves at farm II were housed individually in pens for a 3 month period.

### *Campylobacter* spp. isolation, identification and quantification

All samples were analysed individually. The samples were transferred to the laboratory in a refrigerated bag at 4°C and analysed immediately. Thermophilic *Campylobacter* spp. were isolated by both, direct plating on modified charcoal cefoperazone deoxycholate agar (mCCDA; Liolfilchem, Roseto degli Abruzzi, Italy), and selective enrichment in Bolton broth (Oxoid, Basingstoke, UK).

To detect campylobacters, portions (10 g) of each faecal sample were diluted with 90 ml buffered peptone water (BPW; Oxoid) and mixed for 1 min. For the enumeration of *Campylobacter* spp., serial 10-fold dilutions of faecal samples were plated directly onto mCCDA. Inoculated mCCDA plates were incubated microaerobically (85% nitrogen, 10% carbon dioxide and 5% oxygen) generated by Campygen (Oxoid) at 37°C for 48 h. After incubation, colonies of campylobacters were counted on the basis of colony morphology and typical cell motility (phase-contrast microscopy). Oxidase test was used for primary confirmation of isolated *Campylobacter* spp. Five putative *Campylobacter* spp. colonies (per faecal sample) were subcultured onto blood agar plates (Blood Agar Base No. 2; Liolfilchem) supplemented with 5% Laked horse blood and incubated at 37°C for 48 h under microaerobic conditions as described above. The purified isolates were subsequently stored at –80°C in BHI broth (BHI; Oxoid) with 30% glycerol (Stanlab, Poland).

A selective enrichment procedure was performed for detect of low numbers of thermophilic campylobacters in faecal samples. For this procedure, 1 g faeces was placed in a tube containing a 9 ml Bolton selective enrichment broth (Oxoid) with Bolton broth selective supplement (Oxoid) and 5% Laked horse blood (Oxoid). Enrichment tubes were incubated microaerobically at 42°C for 24 h. After incubation, 10 μl of the enrichment culture was streaked onto mCCDA plates. The identification and purification of *Campylobacter* isolates was further performed as described above. *Campylobacter* counts (cfu/g) of the faecal cattle samples were calculated according to ISO 10272–2:2006.

*Campylobacter* spp. DNA was extracted from presumptive colonies using the boiling method. Briefly, after growing the bacteria on blood agar plates, a loopful (~10 μl) of bacterial culture was taken from two days incubated blood agar plates supplemented with 5% horse blood. The cells were transferred to an Eppendorf tube containing 500 μl distilled water. The samples were vortexed. The suspension was heated at 100°C for 10 min and then centrifuged for 5 min at 14 000 rpm. The supernatant was transferred into a new tube. Extracted DNA was used immediately for PCR amplification or stored at -20°C until examination.

*Campylobacter* isolates were identified to the species level by a multiplex PCR assay described by Wang et al. (2002) with minor modifications. *Campylobacter* spp. (23S rRNA) *C. jejuni* (*hip*O), *C. coli* (*gly*A), *C. lari* (*gly*A), *C. upsaliensis* (*gly*A) and *C. fetus* subsp. *fetus* (*sap*B2) primer mix was used to identify the species [24].

Each PCR mixture contained 2.0 μl of a 2 mM deoxynucleoside triphosphate mixture, 2.5 μl of 10X reaction buffer, 2.5 μl of 25 mM MgCl2, 0.25 μl of HotStart Taq DNA polymerase (MBI, Fermentas), 1 μl of a 100 μM primer mixture containing 23S rRNA (0.5 μM), hipO (1 μM) and glyA (0.5 μM) primers, 1 μl of chromosomal DNA, and MiliQ water to a final volume of 25 μl. DNA amplification was carried out in a thermocycler using an initial denaturation step at 95°C for 6 min followed by 30 cycles of amplification (denaturation at 0.5 min, annealing at 53°C for 0.5 min, and extension at 72°C for 0.5 min), ending with a final extension at 72°C for 7 min. Each PCR product (11 μl) was loaded into a 1.3% TopVisionLM GQ Agarose (MBI, Fermentas) gel wells containing 0.05 μl/ml of ethidium bromide solution and analyzed by gel electrophoresis. The gel was visualized on an UV board. The GeneRulerTM 100 bp DNA Ladder (MBI, Fermentas) was used as the molecular size marker.

### Genotyping of *C. jejuni* isolates

*C. jejuni* isolates were selected according to farms and dairy cattle age. Overall 49 isolates were genotyped. After DNA extraction, *fla*A PCR-RFLP genotyping was performed on *C. jejuni* isolates according to the technique described previously [25]. Primers A1 5’-GGA TTT CGT ATT AAC ACA AAT GGT GC-3’ and A2 5’-CTG TAG TAA TCT TAA AAC ATT TTG-3’ were used to amplify the *fla*A gene from *C. jejuni*. The restriction enzyme *Hpy*F31 (*Dde*I) (ThermoScientific, Waltham, US) was used for the RFLP analysis of the PCR product. The GeneRuler 100 bp plus DNA Ladder (ThermoScientific) was used as the molecular size marker. *fla*A types were assigned manually by comparing band positions.

### Statistical analysis

Obtained data were analysed with SPSS 16.0 software with analysis of variance using the General linear model (GLM) procedure. A chi-squared (χ^2^) test was used to compare the prevalence of *Campylobacter* from different farms or cattle age groups. Differences were considered statistically significant when p≤0.05. The Simpson’s index of diversity (D) was used to determine the genetic diversity of *C. jejuni* genotypes [26]:

$$D=1-\frac{1}{N\left(N-1\right)}\sum_{j=1}^{s}\mathrm{nj}\left(\mathrm{nj}-1\right)$$

N - number of isolates tested;

S - number of different genotypes;

nj - number of isolates belonging to type j.

## Results

### *Campylobacter* prevalence

In this study, *Campylobacter* spp. were isolated from 157 (78.5%) out of 200 faecal samples collected from three dairy cattle farms located in the central part of Lithuania (Table 1). Of these, 14 samples (8.9%) were confirmed positive only after an enrichment step, whereas 143 samples (91.1%) were confirmed positive after direct plating, suggesting a high number of *Campylobacter* in dairy cattle faeces. Dairy cattle herds of all three farms were *Campylobacter* spp. positive, with a prevalence ranging from 75% (farm I), 77.5% (farm II) to 83.3% (farm III). The individual farm had no significant influence (p < 0.05) on the prevalence of this pathogen. When combining data of all three farms, the prevalence of *Campylobacter* spp. was highest among calves (86.5%) and heifers (86.2%), whereas only 60.6% of dairy cow samples contained campylobacters. The highest *Campylobacter* spp. prevalence was found in calves faecal samples collected at the farms I and III, with 89.4% and 100%, respectively. However, differently from farms I and III, heifers from the farm II were more frequently (p < 0.05) infected than calves and cows. *Campylobacter* spp. bacteria were equally prevalent among calves and cows at farm II (p > 0.05).

**Table 1:** ***Campylobacter *** **spp. prevalence, number and species distribution in the dairy cattle farms**

**Source**	**Age group**	**Prevalence (%) (pos. samples/no. of samples tested)**	**Quant. load (log**_**10 **_**cfu/g) (Mean ± SD)**	**Positive samples No./%**				
				***C. jejuni***	***C. coli***	***C. lari***	***C. fetus *** **subsp. *fetus***	***C. *** **spp.**
Farm I	Calves	89.4^a*^ (17/19)	5.62^a*^ ± 0.95	7/41.2	4/23.5	-	4/23.5	3/17.6
	Heifers	85^b^ (17/20)	4.37^b^ ± 0.54	13/76.5	1/5.9	2/11.8	3/17.6	1/5.9
	Cows	53.2^c^ (11/21)	3.55^c^ ± 0.92	11/100.0	3/27.3	-	-	-
Farm II	Calves	70^a^ (14/20)	4.64^a^ ± 1.29	6/42.9	1/7.1	1/7.1	4/28.6	3/21.4
	Heifers	85^b^ (34/40)	4.48^b^ ± 0.69	21/61.8	13/38.2	2/5.9	1/2.9	5/14.7
	Cows	70^a^ (14/20)	4.17^c^ ± 0.54	7/50.0	5/35.7	-	6/42.9	1/7.1
Farm III	Calves	100^a^ (20/20)	5.49^a^ ± 0.90	14/70.0	5/25.0	-	-	5/25.0
	Heifers	90^b^ (18/20)	4.82^b^ ± 0.83	17/94.4	5/27.8	-	-	3/16.7
	Cows	60^c^ (12/20)	3.29^c^ ± 0.44	8/66.7	1/8.3	-	1/8.3	5/41.7
**Total**		78.5% (157/200)	4.5 ± 1.03					

*-Numbers followed by a different letter in the column are significantly different (p < 0.05) for different age groups the individual farm.

Three *Campylobacter* species (*C. jejuni*, *C. coli*, *C. fetus* subsp*. fetus*) were found in samples collected from all sampled farms (Table 1), whereas *C. lari* species was detected in faecal samples collected at the farms I and II. The most prevalent species was *C. jejuni* (66.2%)*,* followed by *C. coli* (24.2%). However, more than one *Campylobacter* spp. species was found in 21.7% of samples.

### Quantitative load of campylobacter

The average count of *Campylobacter* spp. detectable in faeces samples was 4.5 log_10_ cfu/g and numbers of bacteria in the faecal samples were not significantly different in all three farms (p > 0.05) (Table 1). Cattle age is an important factor influencing the number of campylobacters in faecal samples, as significant differences were found among all three cattle age groups (p < 0.05). The highest numbers of *Campylobacter* spp. were found in faecal samples of calves (average 5.3 log_10_ cfu/g), whereas cow samples harboured the lowest number of *Campylobacter* spp. (average 3.7 log_10_ cfu/g).

### Genotype diversity of *C. jejuni* isolates

The *fla*A PCR-RFLP typing of 49 *C. jejuni* isolates resulted in 19 different *fla*A types (Table 2). Genotypes III, VI and XVII were found in samples of all three farms. Genotype III was dominant throughout all three dairy cattle farms. *C. jejuni* genotype I was dominant in calves samples whereas genotype III in young cattle samples, respectively. In addition, genotyping results revealed that several genotypes co-existed in each farm. Several genotypes were specific for an individual cattle age group (Table 2). Only one genotype (genotype V) was identified among all cattle age groups samples collected at the farm II. Genotype VII was dominant in cow samples. The highest diversity of *C. jejuni* genotypes was found at farm II (D = 0.92), whereas the lowest diversity was detectable at farm III (D = 0.75) (Table 2). Isolates from cows samples showed the highest genetic diversity (D = 0.93), while the lowest diversity of the genotypes was identified among isolates from calves (D = 0.76).

**Table 2:** **Distribution and diversity of *C. jejuni fla*** **A genotypes among different farms and cattle age groups**

***fla*** **A types**	**Absolute no. of isolates per *fla*** **A type**	**No. of isolates per *fla*** **A type**		
		**Farm I**	**Farm II**	**Farm III**
I	9	-	-	7A*; 2B
II	4	3B	1C	-
III	9	1A; 3B	2B	1A; 2B
IV	1	1C	-	-
V	4	-	1A; 2B; 1C	-
VI	3	1A	1B	1B
VII	3	-	-	3C
VIII	1	-	1H	-
IX	1	1B	-	-
X	1	-	1B	-
XI	1	1B	-	-
XII	1	-	1C	-
XIII	2	1A	-	1A
XIV	1	-	-	1C
XV	1	-	1B	-
XVI	1	1C	-	-
XVII	4	2B	1B	1C
XVIII	1	-	1B	-
XIX	1	1A	-	-
**Total**	49	16	14	19
Simpson's Index (D)		0.91	0.92	0.75

*Source of isolate: A - calves; B - heifers; C - cows.

## Discussion

To our knowledge, this is the first study investigating the *Campylobacter* prevalence and quantitative load in dairy cattle in the Baltic States. Recent studies have shown that the contribution of non-poultry associated *Campylobacter* strains to human campylobacteriosis is considerable [8,27].

Despite the fact that *Campylobacter* is common in cattle herds, our study revealed a very high prevalence of these bacteria (average 78.5%) in all 3 farms. Most other comparable studies reported prevalences between 5% and 67.1% [10-14,18,22,23,28-32]. Since these studies vary in sampling design, culture methods and conditions, a direct comparison of the results is difficult. However, our data contribute to previous discussions that cattle are significant reservoirs for *Campylobacter* spp. and could be a source of infection for other animals and humans [5,14]. There are studies describing transmission of campylobacters from cattle to poultry production chain. The significance of *Campylobacter* colonization of cattle are related not only to the potential for contamination of milk at the farm and the carcass at slaughter, but also surface and sub-surface water. In addition, several studies have found the presence of cattle, on broiler farms is associated with increased risk of infection in broiler flocks [6,21].

Results of several studies are contradictory, regarding the effect of age on the prevalence of *Campylobacter* in dairy cow farms. Our study showed that the cattle age significantly influences the prevalence of *Campylobacter* spp. (p < 0.05): the highest prevalence was observed in the calve groups in comparison to milking cow groups in farms I and III then animals are kept in groups of about 10–20. Similarly, former studies concluded that calves became colonized with *Campylobacter* within 4 days, with maximal *Campylobacter* shedding occurring at 1–2 months of age with prevalences of up to 42.1-46%, while *Campylobacter* prevalences among older cows were significantly lower 9.2-28.5% [12,14]. However, a more recent study [15] argued that dairy cattle age did not influence the prevalence of campylobacters in cattle faeces and *Campylobacter* prevalence between age groups ranged from 35% in animals above 60 months of age to 50% in those below 30 months. However, in this study the difference in prevalence between age groups was not significant. It should be mentioned that the prevalence of campylobacters among calves at the farm II was significantly lower in comparison to the prevalence among the corresponding age group calves at the farms I and III. This could be explained by different housing systems, since calves (also heifers and milking cows) in farm I and III were kept in groups of 10–20 animals, whereas calves at farm II were kept individually. One infected calve can contaminate the environment what leads to a quick transmission of campylobacters among calves of the same group [33].

Our study showed that *C. jejuni* was the dominant species in the tested samples, followed by *C. coli*. This is in accordance with other studies, which describe *C. jejuni* as the dominant *Campylobacter* species in cattle intestines [11,28]. However dominance of *C. jejuni* can differ at the broad range as Wesley et al. (2000) and Nielsen (2002) have reported prevalence of *C. jejuni* from 7% to 38% in dairy herds, which are at least twice lower in comparison to 66.2% prevalence revealed by our study. So we could speculate that dairy cattle play a significant role in *C. jejuni* epidemiology (responsible for 90% of human campylobacteriosis cases) as an important host of *C. jejuni*[3]. In addition, our study showed that cattle age is a significant risk factor for quantitative load of *Campylobacter* spp. Calves showed the highest numbers of *Campylobacter* in faeces, followed by heifers in all three farms. Cows had the lowest *Campylobacter* load in faeces. This is in agreement with other studies, demonstrating a similar dependence on higher concentrations in younger animals [14,34]. Overall, our quantitative data (4.5 log_10_ cfu/g) are comparable to previously published results, showing concentrations of 3.7 log_10_ cfu/g [14] and 4.4 log_10_ cfu/g [32].

By applying the *flaA* PCR–RFLP method, which is widely used for genotyping of campylobacters, a high strain diversity was identified in the *C. jejuni* strains isolated at three dairy cow farms (Table 2). Multiple genotypes on the same farm may be related to multiple sources of infection or to a persistent infection leading to genetic variations within the *C. jejuni* population. Oporto et al. (2007) found a similarly high *C. jejuni* genetic diversity in dairy cattle (12 *flaA* types from 43 isolates) using the *flaA* PCR-RFLP method. Similarly, nine to 35 *flaA*-types were identified among cattle isolates in other studies [35,36]. In conclusion, although the overall results suggest that some genotypes exist in all dairy cattle farms, more than half of the genotypes in each farm were specific to the individual farm. This may be due to the fact that the geographical location has an influence on *C. jejuni* genetic diversity.

## Conclusions

This study revealed a high prevalence and quantitative load of *Campylobacter* spp. in calves, heifers and milking cows at the three dairy farms, supporting the significance of cattle as a potential reservoir of transmission of *Campylobacter* spp. to humans. Despite the fact that age is the significant factor influencing the prevalence of campylobacters among calves, heifers and milking cows, our finding suggest that healthy dairy cattle of any age group can play a significant role in the contamination of the environment and the possible entrance of *Campylobacter* spp. into the food chain. Several different *C. jejuni* genotypes observed in each farm indicate multiple pathways involved into colonisation of dairy herds by *Campylobacter* spp. Further studies are needed to investigate the entrance pathways of *Campylobacter* into the herds which could lead to the development of specific measures to reduce colonisation of cattle with *Campylobacter* spp.

## Competing interests

The authors declare that they have no competing interests.

## Authors’ contributions

SR collected and analysed the data, did the literature review and drafted the manuscript. AR, AM and ET assisted with data collection and testing. TA took part in the writing. MM generated the study design and revised the manuscript. All authors read and approved the final manuscript.
